# The research on 3D printing fingerboard and the initial application on cerebral stroke patient’s hand spasm

**DOI:** 10.1186/s12938-018-0522-4

**Published:** 2018-06-26

**Authors:** Kai Wang, Yiting Shi, Wen He, Jing Yuan, Yuan Li, Xiaolin Pan, Cuilian Zhao

**Affiliations:** 1 Department of Rehabilitation Medicine of Shanghai Fourth Rehabilitation Hospital, Shanghai, 200041 People’s Republic of China; 2 Shanghai Oumu Health Management Consulting Co. Ltd., Shanghai, 200041 People’s Republic of China; 3 Department of Mechanical Engineering and Automation, Shanghai Univ., Shanghai, 200072 People’s Republic of China

**Keywords:** 3D printing, Fingerboard, Auxiliary brace, Cerebral stroke, Spasm

## Abstract

**Purpose:**

To research the possibility of designing customized 3D printing fingerboard to apply to the limb rehabilitation of cerebral stroke patients as well as the prevention and treatment of finger spasm, through 3D printing technology.

**Methods:**

Taking 18 hospitalized cerebral stroke patients for example, through scanning, molding and printing, to make and wear 3D printing fingerboard for them, and then observe the compliance, main complaint, muscular tension of affected hand and changes on range of motion after they wear the fingerboard for 3 weeks and 3 months.

**Results:**

Have acquired completed data from 13 patients. The time of them wearing the fingerboard every day varied from 1 to 8 h, and most of them reflected that they felt comfortable and there was no feeling of worsened pain or finger skin allergy. In addition, the patients’ grip strength, hand function and range of motion improved by varying degrees while their muscular tensions declined by varying degrees. The tension and bending resistance of the fingerboard all met the patients’ treatment requirements.

**Conclusions:**

With the advantages of being accurate and customized, 3D printing fingerboard can benefit patients fixing and orthopedic treatment, and even prevent and treat cerebral stroke patient’s finger spasm.

*Trial registration* The research topic has been registered in Chinese Clinic Trial Registry. Registration time: January 15, 2016. Registration topic: The Use of 3D Printing Technology in the Orthotic of Extremity Rehabilitation of Stroke Patient. Registration Number: ChiCTR-INR-16007774

## Background

In terms of the application of traditional fingerboard on cerebral stroke patients, there have been defects such as inaccurate size and less customized design, which results in the fact that patients can’t wear it comfortably and that the treatment can hardly be continuous. As the development of 3D printing technology, its application on rehabilitation auxiliary brace becomes possible [1–3]. Theoretically, 3D printing fingerboard can treat cerebral stroke patient’s hand-spasm more effectively since it is more customized and more comfortable to wear. However, further research should be done on whether this technology is reliable and whether the fingerboard can meet the requirement of clinic rehabilitation treatment.

## Methods

### Example patients

Taking 18 cerebral stroke patients hospitalized in our hospital during January to October 2016, whose conditions were in accordance with the cerebrovascular disease diagnostic criteria set on the 4th National cerebrovascular disease seminar in 1995 and verified by head CT or MRI.

Meanwhile the patients have met all of the following inclusion conditions.

1. Age: 40–80; disease duration: 2 weeks–3 months.
2. After getting sick, the patients didn’t have cognition or affective disturbance, no severe organ dysfunction.
3. Willing to accept rehabilitation trainings during hospitalization.
4. Patient’s hand muscle tension was increased and graded above level two by Modified Ashworth Scale (MAS).
5. Patients or family members were willing and able to sign the informed consent. Patients were willing to accept follow-up visit during the study.

### The making of 3D printing fingerboard

#### Assessment and prescription

##### Assessment

A. Muscular tension: To respectively assess the muscular tension of extensor and flexor of five metacarpophalangeal joints and all interphalangeal joints of the affected hand.
B. Range of motion: To respectively assess the range of motion of active and passive of five metacarpophalangeal joints and all interphalangeal joints of the affected hand.
C. Measure of hand shape: To measure the distances from the top of thumbs, middle fingers and little fingers on both hands to the midpoint of transverse carpal ligament, and to assess whether both hands are symmetric.

##### Prescription

According to the assessment results, doctors make a prescription of 3D printing fingerboard based on biomechanics and neurophysiology theories.

A. According to the assessment of the range of motion of active and passive joints on the affected hand, to confirm the bending angles of metacarpophalangeal joints and interphalangeal joints of the healthy hand, and to make the palm mold through the healthy hand.
B. According to the location of bending spasm joints on the affected hand and the three-point pressure theory of the orthotics, to confirm the location of the orthopedic bandages in fingerboard.

#### The making and scanning of palm mold

**Fig. 1:**
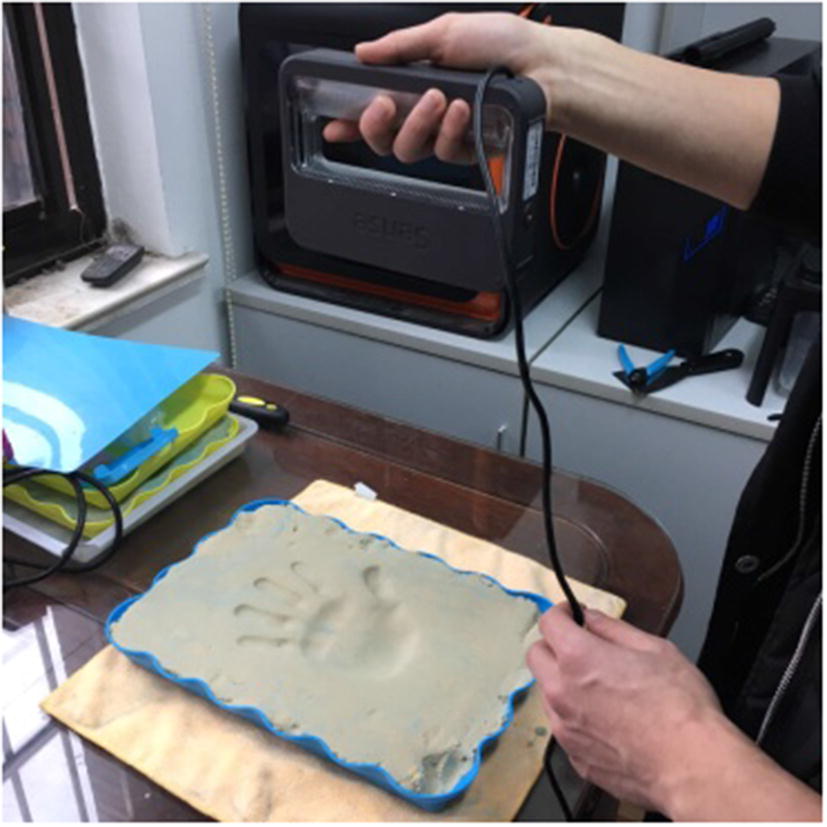
Making and scanning of palm mold

We make a flat mold by using soft sand, put patients’ healthy hand on the mold, and extrude a palm model based on the requirements of Prescription A. Then, we scan with a hand-held optics 3D scanner and generate the palm molding file (STL format) (Fig. 1).

#### The process of palm molding file

We import the palm molding file (STL format) into Netfabb software to renovate the reverse triangular face, incorrect side and hole that appear. We do smoothing process to the surface structure of the triangular face. The smoother the surface is, the more convenient the making of the following 3D fingerboard file will be.

#### The making of 3D fingerboard file

We import the processed palm molding into 3D Max, and generate 3D fingerboard molding file based on clinic requirements, the procedures are as follows.

##### Shape processing

**Fig. 2:**
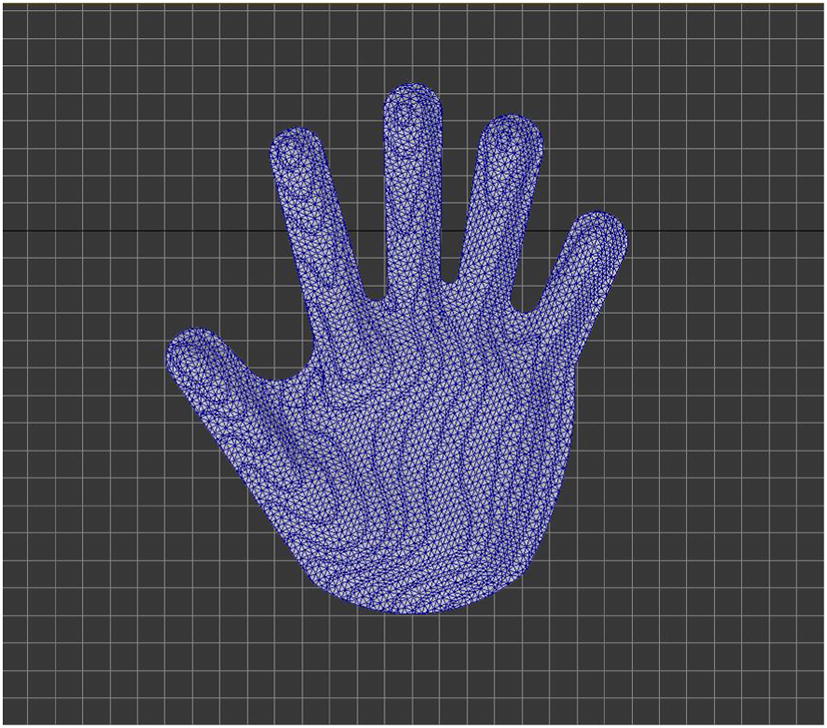
Confirm the shape of fingerboard

A. Mirror symmetry processing: If both hands are symmetric, then turn the palm molding acquired from the healthy hand to the affected hand molding by mirror processing. If not, to do some adjustments based on the measure results after mirror symmetry processing.
B. Outline renovate: To confirm the shape of fingerboard (Fig. 2).

##### The making of holes

The function of holes is to let through the ligation bandage, and the ligation bandage can fix the hand on the fingerboard. The ligation bandage is divided into fixing type and orthopedic type.

**Fig. 3:**
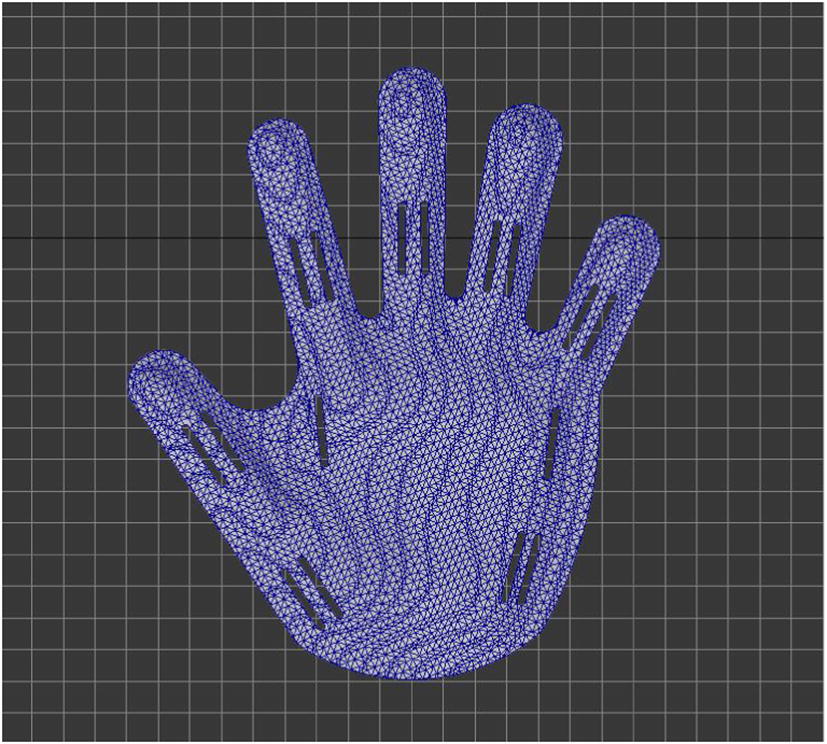
Making of holes

A. The holes that fixing bandage needs: are used for letting through the fixing bandage, one for fixing wrist joints and one for metacarpophalangeal joints. The width of holes is 2 mm and the length 25 mm.
B. The holes that orthopedic bandage needs: The design of the holes of orthopedic bandage depends on the degree and part of the bending spasm of patient’s fingers. Based on the three-point oppressing theory of orthotics, the orthopedic bandage that crosses the holes can correct the flexion contracture of interphalangeal joints and metacarpophalangeal joints. The width of holes is 2 mm and the length 25 mm (Fig. 3).

##### Overall duplication

**Fig. 4:**
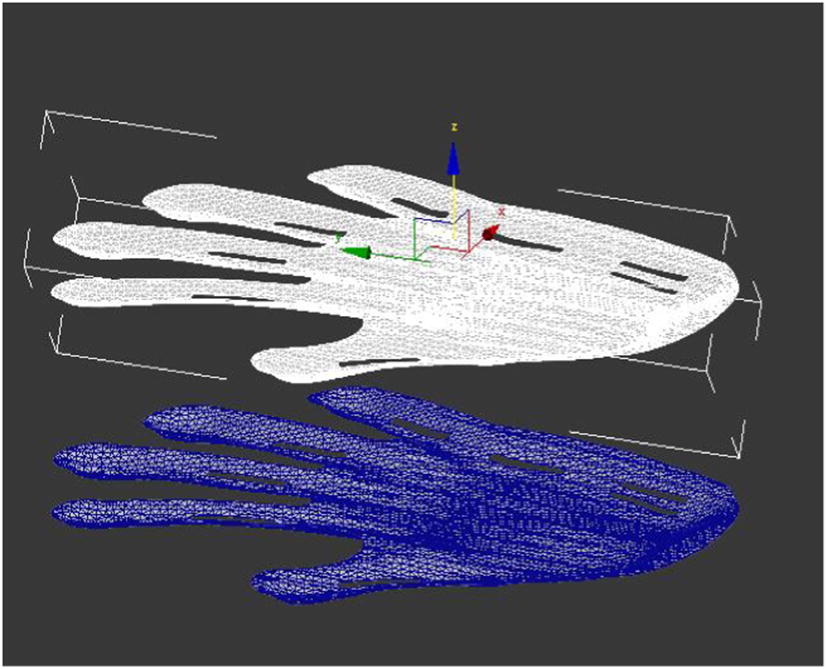
Overall duplication

To duplicate the above completed molding to two pieces, one for processing and one for standby (Fig. 4).

##### Shirr processing

**Fig. 5:**
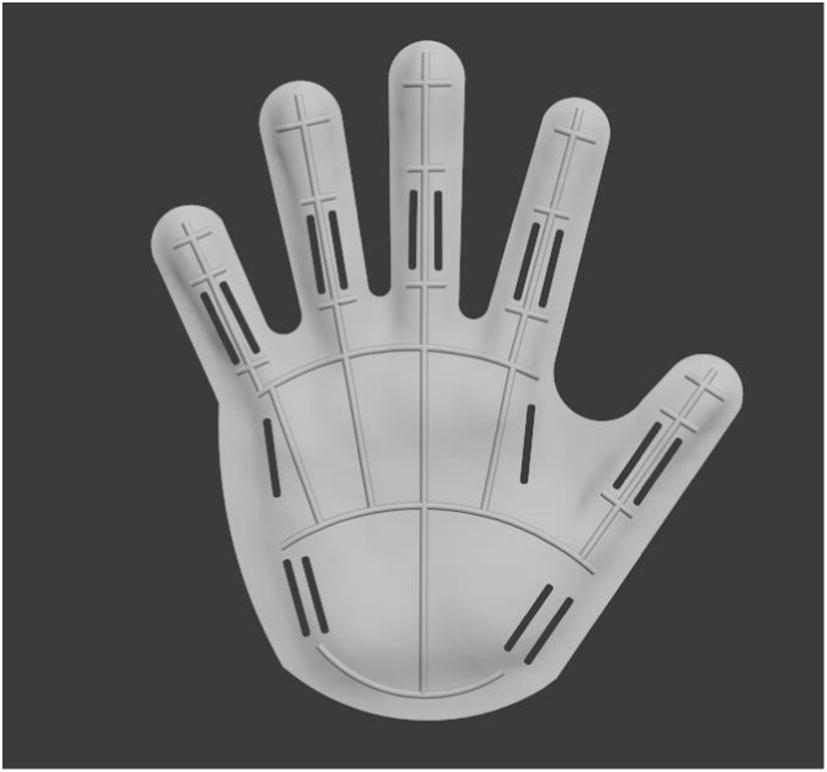
Shirr processing

According to the mechanics theory, to conduct shirr processing to the unprocessed palm molding with the purpose of increasing the bending resistance capability of the palm (Fig. 5).

##### Extrusion and weld

**Fig. 6:**
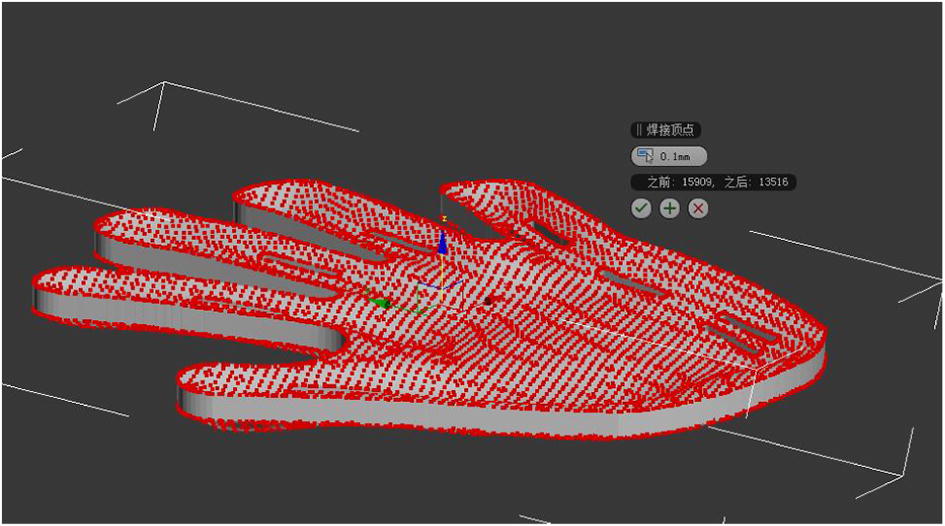
Extrusion and weld

After conducting shirr processing, to extrude a 3 mm space on the reverse of the molding to weld with the standby one, and then to repair the loopholes left by weld. At this stage, the 3D fingerboard molding is basically completed (Fig. 6).

##### Hole processing

**Fig. 7:**
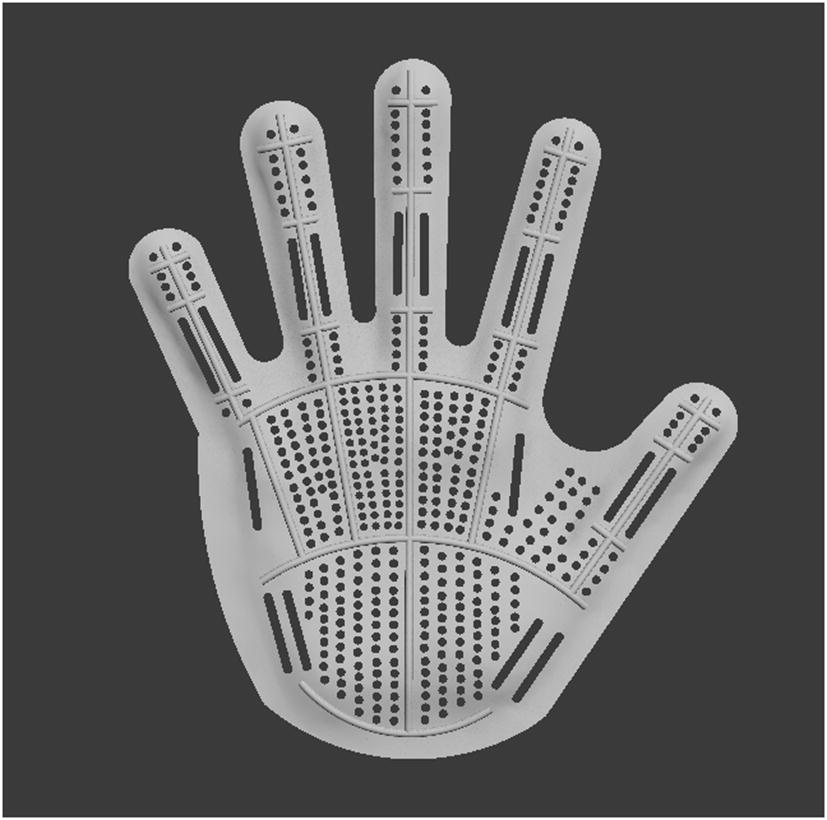
Hole processing

Open holes on the palm and finger part of 3D fingerboard, to make the patients feel ease and ventilated when wearing it and to improve the patients’ comfort level, but the hole processing can’t decrease the intensity of fingerboard (Fig. 7).

Finally export the completed 3D fingerboard molding file in STL format to the 3D printer section software.

#### Selection of printing materials and methods

To select FDM (Fused Deposition Modeling) printer. The 1.75 mm PLA material is used for printing. To use Cura section software that affiliated to the printer to section and adjust the optimized setting of printing method, and the printer process lasts around 7 h. When printing is finished, to remove the backing material.

#### Fine processing to the completed brace

After the backing material is removed, the surface of the fingerboard is comparatively rough, then to do labor polish with silica sand paper. Considering patients’ safety and comfort, to smooth the edges of two sides on the brace in order to avoid the patients being bruised. To rounding the edge of holes in order to enable the ligation bandage can move smoothly when the patients are wearing it.

**Fig. 8:**
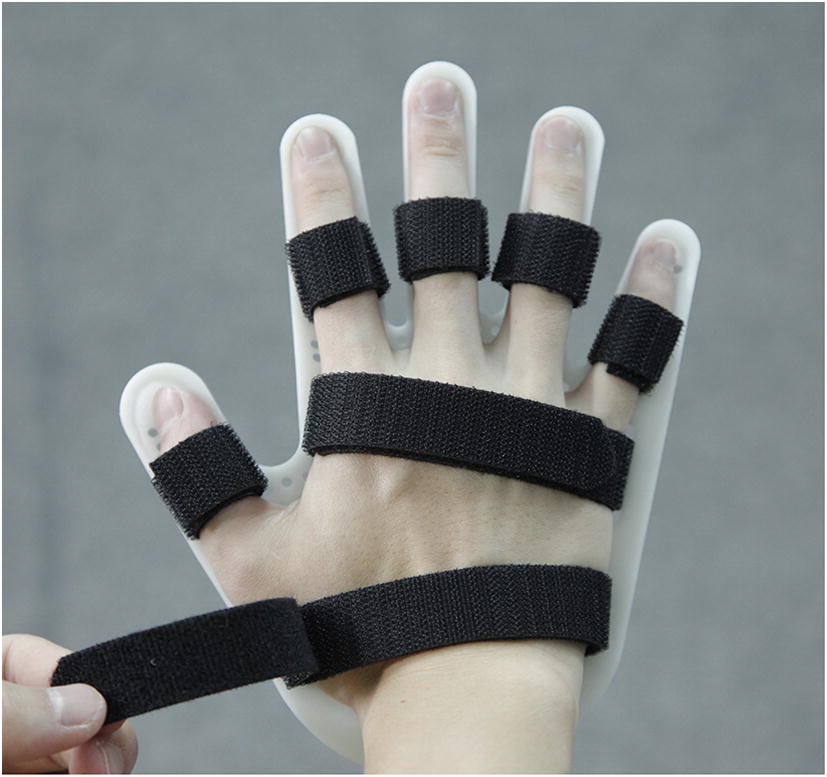
Fixing and orthopedic treatment to patients’ fingers

To use the 2 cm-width one-side smooth sticking band to cut into a proper length according to the width of patients’ finger joints and palms, to cross the holes, to wear the brace on their hands, and to complete fixing and orthopedic treatment to patients’ fingers, palms and wrists based on the requirements of prescription (Fig. 8).

### Observation methods

The 18 hospitalized cerebral stroke patients all met the selected standards. One week later, after signing the informed consents, they wore customized 3D printing fingerboard. They were required to wear the fingerboard after daily regular rehabilitation training, averaging 2 h in the morning, afternoon and evening. They could leave the hospital after wearing the fingerboard for 3 weeks, but they needed to keep wearing it if they went back to community or went to other hospitals. We recorded patients’ wearing time of the brace and their comfort levels, assessed the muscle force, gripping power, range of motion, muscular tension, pain and allergy of the affected hands. The assessment should be done each time before wearing, after 3 weeks and after 3 months. If the patient could not go to the hospital to receive the assessment, then the family’s main watcher should be contacted by phone or home assessment should be done.

### Evaluation method

#### Hand pain

Hand pain of the affected hand was evaluated by using Visual Analogue Scale (VAS) method. The evaluation was performed by using the VAS card, supervised and manufactured by Chinese Association for the Study of Pain, shows a series of numbers moving from “pain-free” (0) to “the most severe pain” (10). We asked patients in pain which number matched up with what they were feeling.

#### Hand grip strength

The grip strength of the affected hand was evaluated by using Grip Strength Index, Grip Strength Index = hand grip (kg)/weight (kg) * 100. The measurement was performed by using hand grip dynamometer. Before taking measurements the patients were requested to stand in a comfortable position with their upper limbs hanging normally beside their bodies. Adjust the handle of the dynamometer to fit the patients’ hand width. The patients were asked to squeeze the dynamometer as hard as possible. The measurement was performed three times, and the reading was taken from the maximum grip scale.

#### Range of motion of hand joints

The range of motion of affected hand joints was measured in the active and passive activities of the patient. The readings of the shortest distance from the fingertip to the palm print, and the flexion and extension angle of the wrist joint, reflecting the range of motion of affected hand joint.

#### Muscle tension of the hand

The muscle tension of patient’s hand was evaluated by using Modified Ashworth Scale (MAS) which was proposed in 1964 by Ashworth and revised in 1987 by Bohannon to assess muscle tension in limbs. It is the most widely used method of measuring muscle spasticity in the world. The scale scores are 0, 1, 1+, 2, 3, 4, the greater the value, the higher the muscle tension.

#### Hand movement function

The movement pattern of the hand was evaluated by using Brunnstrom approach. This method was proposed in the 1960’s by Signe Brunnstrom, a physical therapist from Sweden. Brunnstrom approach sets out 1–6 levels reflecting the changes in the movement patterns of extremities in patients with upper motor neuron disease, the greater the level, the closer the motion mode of the hand to normal.

### Statistical method

Use SPSS Version 20 software to conduct data statistics. For three-group comparison of measure data, use one-way ANOVA; for three-group comparison of counting data, use Wilcoxon signed rank test. The P < 0.05, difference has statistical significance.

## Results

### Patients’ conditions

18 patients wore the fingerboards and received an assessment after 3 weeks. 3 patients didn’t keep wearing to the end, among which 1 patient had depression and refused any rehabilitation treatment, 1 patient got shoulder-hand syndrome, feeling swelling on hand and refusing to wear, the other 1 patient felt that the spasm alleviated and didn’t wear anymore; in the assessment done after 3 months, 1 patient didn’t wear since the cerebral stroke recurred, 1 patient lost contact and the other 13 patients gave completed data. No report of breaking brace appeared during the observation.

Among the 13 patients who completed the entire research, there are eight males and five females; average age 68.3 ± 4.9; one had cerebral hemorrhage, ten had cerebral infarction, two had cerebral infarction and hemorrhage; six had left-side hemiplegia and seven had right-side hemiplegia; average days of attack 60.9 ± 23.5; except for one who didn’t had complication, other 12 had hypertension, diabetes, coronary disease, atrial fibrillation and other disease, but they were stable under medicine control. Before wearing, the hand function (Brunnstrom level) of 13 patients varied from 2 to 4, the flexor muscular tension of metacarpophalangeal joint (Ashworth level) varied from 2 to 3. 6 patients had mild swelling on hand, 5 patients felt mild pain on hand and 1 patient felt strong pain. Refer to Table 1.

**Table 1:** 13 cerebral stroke patients’ conditions

No.	Gender	Age	Education background	Disease time	Disease position and character	Complication	Hand function level (Brunnstrom)
1	M	73	High school	43	Left temporal occipital large area infarction	Hypertension, arrhythmia	3
2	F	64	College	86	Left basal ganglia infarction	Diabetes type 2, coronary disease, atrial fibrillation	2
3	F	76	Middle school	67	Left ventricle side infarction	Coronary disease, diabetes type 2, hypertension, stress ulcer	2
4	M	61	High school	34	Left basal ganglia and centrum semiovale infarction	Hypertension, thyroid nodule	2
5	M	61	Middle school	57	Left forehead, temporo and parietal lobe infarction with hemorrhage	Hypertension, coronary disease, atrial fibrillation, secondary epilepsy	2
6	F	67	High school	63	Leftmultiple cerebral infarction	Hypertension, surgery on left lung cancer	2
7	F	68	Middle school	23	Right temporo and basal ganglia infarction	Coronary disease, arrhythmia	2
8	M	68	Middle school	82	Left frontal lobe and basal ganglia cerebral hemorrhage	No	3
9	M	68	College	84	Right basal ganglia and frontal lobe infarction with hemorrhage	Hypertension	4
10	F	76	Primary school	49	Right basal ganglia infarction	Diabetes type 2	3
11	M	68	College	86	Right frontal lobe, left parietal lobe and basal ganglia infarction	Diabetes type 2	3
12	M	72	College	30	Brain stem infarction	Coronary disease	4
13	M	66	Middle school	88	Right frontal lobe infarction	Hypertension, diabetes type 2	4

### Conditions of patients’ wearing 3D printing fingerboards

In the follow-up visit during the process that patients wore fingerboards for 3 weeks and 3 months, the time of wearing was between 1 and 8 h, averaging 4.54 and 4.77 h respectively. Most of the patients were satisfied with the comfort when wearing and had no feeling of extra pain. For patients needed three-point oppressing orthopedic treatment on finger spasm, they could receive the oppressing from orthopedic bandage. No patient had finger skin allergy; no patient was reported to have hand new swelling, the former hand edema patients’ symptoms didn’t get aggravated, and the symptoms became alleviated as the rehabilitation treatment went on.

After 3 months of rehabilitation and wearing fingerboards therapy, the grip strength index of patient’s hand increased, suggesting the increase of hand holding and grasping. The shortest distance between the fingertips of the middle finger and the palm print of the hand was shortened in the active and passive activities of the patient, the flexion and extension angle of the wrist joint was improved in the active and passive activities of the patient, reflecting that the range of motion of the patient’s hand joints was increased by varying degrees. The Brunnstrom levels of the patient’s hands was increased, suggesting that the movement patterns of the hands were improved, which meant that the function and flexibility of the patient’s hands were improved. The MAS grading of flexor muscles of the metacarpophalangeal joints was decreased by varying degrees during passive stretching, suggesting that the muscle tension of the patient’s hand declined by varying degrees. However, due to the small number of cases in the overall statistical observation, there was no significant difference from statistical level, except hand movement mode and hand muscle tension change, it is meaningful for wearing for 3 months compared with before wearing. Refer to Table 2.

**Table 2:** Conditions of 13 cerebral stroke patients’ wearing 3D printing fingerboards

	Before wearing					3 weeks					3 months				
Daily wearing time (h)	0					4.54 ± 2.60					4.77 ± 2.24				
Pain degree (VAS)	2.0 ± 1.58					1.85 ± 1.34					1.69 ± 1.03				
Skin (edema, allergy)	6 mild edemas					5 mild edemas, 1 swelling, no allergy					3 mild edemas, 1 swelling, no allergy				
Gripping power index	0.018 ± 0.041					0.025 ± 0.054					0.032 ± 0.067				
Range of motion															
Shortest distance from the fingertip to the palm print (mm)															
Active	14.02 ± 20.65					12.31 ± 16.07					11.46 ± 15.88				
Passive	6.15 ± 12.79					4.15 ± 10.53					3.85 ± 9.61				
Wrist joints (flexor)															
Active	2.62 ± 8.27					5.08 ± 11.6					5.83 ± 13.8				
Passive	41.2 ± 11.6					45.2 ± 11.3					45 ± 11.2				
Wrist joints (extensor)															
Active	1.0 ± 2.77					3.62 ± 9.83					3.85 ± 11.2				
Passive	35.5 ± 17.1					38.4 ± 15.8					39.8 ± 17.5				
Hand movement mode															
Brunnstrom level	1	2	3	4	5	1	2	3	4	5	1	2	3	4	5
The number of cases	0	6	4	3	0①	0	4	5	4	0	0	1	7	5	0①
Muscular tension (metacarpophalangeal joint, extend)															
MAS	1	1^+^	2	3	4	1	1^+^	2	3	4	1	1^+^	2	3	4
The number of cases	0	0	9	3	1②	0	0	9	4	0	4	3	4	2	0②

①, ② P < 0.05

## Discussion

3D printing technology, also known as rapid proto-typing technology, is a new additive manufacturing technology that stacks materials based on three-dimensional digital model, which is contrary to traditional subtractive manufacturing technology. At this moment, the most common used 3D printing order is to scan the image to get the STL or OBJ file, conduct three-dimensional reconstruction through computer aided design software, import the result into 3D printer, printer each lay, and stack the lays to acquire a physical model. Due to its accuracy, customization and remote control, 3D printing technology has bright development in medical science, including model design, navigation template, biological printing, customized prosthesis, auxiliary brace, etc.

Cook acquired image through 3D scanner, used different materials to make complicated kid’s brace with different thickness and shape, used SLS method to print hard shell and used SLA method to print soft core [2]. Toshev used similar method to research on upper limb brace, scanned the upper limb, cut the limb surface file, imported into CAD to make brace model, and made static customized upper limb brace [3]. 3D printing upper limb brace is easy and convenient to wear, and it fits well with patient’s skin and doesn’t impact the hygiene, patient can even wear it when taking a shower. Different materials have different functions, that is, hard materials can help support and protect patients while soft ones can help patients exercise. Therefore, 3D printing brace is expected to become the main technology in regard to upper limb in the future.

More than 70% of cerebral stroke patients have upper limb damage, spasm and contracture can easily occur on the flexor muscle group of patient’s wrist and hand, if it is not intervened in time, the hand will become fist clenching state, patient’s self-help ability, hand hygiene and life quality will be impacted [4]. There are many methods can help control hand spasm and contracture, but no general consent is acknowledged [5]. Currently, to use long-term brace is the popular treatment method [6].

However, two essays review the practical effect of cerebral stroke patient’s upper limb brace [7, 8], pointing out static brace has no effect in improving upper limb function and range of motion or releasing pain. Many scholars think the research conclusion should be further discussed since it lacks randomized controlled experiment and standard research method, and it only observes the short-term treatment effect of the brace. Aukje Andringa thinks that even though there is no research on long-term wearing, doctors and patients will still believe the brace is effective, because contracture develops slowly and it takes at least 6 months to observe the brace’s effect of preventing contracture. To maintain the range of motion is an effective index of brace’s preventing hand contracture, no increasing muscular tension, spasm, joint deformity and limitation of motion on finger has indicated the brace is effective. The research reflects that for the patient who can bear static brace, to wear brace can help prevent finger contracture [9].

Though there is academic controversy, doctors still suggest patients to wear brace in clinic treatment, however, a large number of patients complain the pain and spasm on hand have aggravated after using it. Because of poor compliance, patients can’t keep wearing the brace for 8 h daily as doctor suggests, which results in limited treatment effect [10, 11]. Not feeling comfortable may be decided by features of the brace, some can only offer limited size and can’t meet the requirements of patient’s palm size or the degree of finger spasm. For the patients who use brace for long term, most scholars think tolerance and comfort level are basic issues that need to be resolved, therefore, customized hand brace is needed to relieve discomfort, including acquiring accurate dissection information, adapting dissection features properly, and avoiding pain and other discomfort from oppressing directly on joints [12]. 3D printing fingerboard meets patient’s demands due to its customized design and accurate making.

There are many research on 3D printing technology of forearm, wrist and hand brace [1–3], the 3D printing fingerboard discussed in this essay includes hand shape scanning, fingerboard molding and 3D printing, the same as the 3D brace making process reported previously. The making process is clear and can be repeated.

1. For the scanning process, we used optics scanner, the affected hand couldn’t place in proper place because of paralysis and spasm. Since most people’s hands were symmetrical, based on image theory, we placed the healthy hand at will, scanned it, and then acquired the information of the affected hand.
2. For molding process, we used 3DMax software. Since the thickness of brace made of thermoplastic plate was normally 2–3.5 mm, we made that of our fingerboard 3.0 mm. We took advantage of 3D molding and did some special handling to the fingerboard: according to individual clinic demand, in order to improve the strength and the pressure capacity of the palm flexion of the fingerboard, and reduce the thickness and the weight of the fingerboard, we increased thickness, added shirr and opened holes on necessary parts. When handling the holes, we paid special attention since the form, size and number of the holes couldn’t reduce the load capacity of the brace. There were professional software engineers handling the molding process and that took around 2 h. It was too professional for a clinic doctor and it would take more time, if there should be a specialized fingerboard software that could be operated without technique requirement, it would be beneficial to the promotion.
3. For printing process, we tried FDM and SLA. We chose ABS, PLA and photosensitive resin as printing materials. We tested the three materials and found the greatest pressure that ABS, photosensitive resin and PLA could bear were 9.7, 37.9 and 14.6 kg respectively, the anti-breaking strength of photosensitive resin was the highest and that of PLA followed; the maximum deflection of fingerboard breaking was 3.5 mm (ABS), 7.2 mm (photosensitive resin) and 2.6 mm (PLA), the breaking character was ductile fracture, and the conclusion was that the fragility of ABS and PLA made fingerboard were high, while the tenacity of photosensitive resin made fingerboard was high. According to the test, all the strength and tenacity of three materials could apply to correct patient’s finger spasm, refer to Ahn and other references for deeper analysis of material character [1]. In this essay, we choose FDM technology that is cheap and PLA material that is biodegradable, which are more convenient for clinic promotion.

In the follow-up visit to the patients who wore 3D printing fingerboard mentioned in this essay, most of them were satisfied when wearing it and no one felt extra pain. During the period, no patient got finger skin allergy or hand swelling, their grip strength, hand function and range of motion improved by varying degrees, while muscular tension declined by varying degrees. There was no fingerboard damaged or broken. Because this research focuses on the process of making 3D fingerboard, whether it is suitable to wear on the hands of patients, and preliminary application observation, the research examples were comparatively few, no randomized controlled, the observation time was short, and the wearing time didn’t reach 8 h per day as the references suggested, which made it hard to observe the significant difference on statistics level of the changes of results except the hand movement pattern and the hand muscle tension 3 months later than before wearing. As for what advantages does this fingerboard have in patient’s recovery of the muscle strength, muscular tension and hand function during rehabilitation process compared to traditional ones, further research will be done.

The 3D printing fingerboard discussed in this essay is worthy of being promoted in clinic practice, since it is customized design, comfortable to wear, good in compliance, will not result in untoward effect and can solve the defects of the traditional finger plate which could neither match hand types nor perform accurate orthopedics. Compared to the traditional products on market, 3D printing brace doesn’t have pricing advantage and is restricted in clinic promotion, because it demands more work and professional software, and the printing materials are limited and expensive. However, as the customized rehabilitation treatment receives more attention and the cost of software, materials and printer decreases, 3D printing brace will be promoted soon in clinic practice.
